# Effects of Separate and Concomitant TLR-2 and TLR-4 Activation in Peripheral Blood Mononuclear Cells of Newborn and Adult Horses

**DOI:** 10.1371/journal.pone.0066897

**Published:** 2013-06-19

**Authors:** Johannes Cornelis Vendrig, Luc Edgar Coffeng, Johanna Fink-Gremmels

**Affiliations:** 1 Veterinary Pharmacology, Pharmacotherapy and Toxicology, Faculty of Veterinary Medicine, Utrecht University, Utrecht, The Netherlands; 2 Department of Public Health, Erasmus MC, University Medical Center Rotterdam, Rotterdam, The Netherlands; Indian Institute of Science, India

## Abstract

Deficient innate and adaptive immune responses cause newborn mammals to be more susceptible to bacterial infections than adult individuals. Toll-like receptors (TLRs) are known to play a pivotal role in bacterial recognition and subsequent immune responses. Several studies have indicated that activation of certain TLRs, in particular TLR-2, can result in suppression of inflammatory pathology. In this study, we isolated peripheral blood mononuclear cells (PBMCs) from adult and newborn horses to investigate the influence of TLR-2 activation on the inflammatory response mediated by TLR-4. Data were analysed in a Bayesian hierarchical linear regression model, accounting for variation between horses. In general, cytokine responses were lower in PBMCs derived from foals compared with PBMCs from adult horses. Whereas in foal PBMCs expression of TLR-2, TLR-4, and TLR-9 was not influenced by separate and concomitant TLR-2 and TLR-4 activation, in adult horse PBMCs, both TLR ligands caused significant up-regulation of TLR-2 and down-regulation of TLR-9. Moreover, in adult horse PBMCs, interleukin-10 protein production and mRNA expression increased significantly following concomitant TLR-2 and TLR-4 activation (compared with sole TLR-4 activation). In foal PBMCs, this effect was not observed. In both adult and foal PBMCs, the lipopolysaccharide-induced pro-inflammatory response was not influenced by pre-incubation and co-stimulation with the specific TLR-2 ligand Pam3-Cys-Ser-Lys4. This indicates that the published data on other species cannot be translated directly to the horse, and stresses the necessity to confirm results obtained in other species in target animals. Future research should aim to identify other methods or substances that enhance TLR functionality and bacterial defence in foals, thereby lowering susceptibility to life-threatening infections during the first period of life.

## Introduction

Due to deficits in both innate and adaptive immune responses, newborn mammals display increased susceptibility to bacterial infections compared with adult individuals [1]. With regard to T helper cell (Th) responses, newborns predominantly display Th2 responses and are deficient in eliciting Th1 responses [1], [2]. In *ex vivo* foal models, basal levels of Th1-related cytokines such as interferon-γ (IFN-γ), tumour necrosis factor-α (TNF-α), and interleukin-6 (IL-6) have been shown to be decreased compared with older individuals [3]–[5]. In addition, monocyte-derived dendritic cells (MoDCs) of foals have been classified as less mature, and express limited levels of IFN-γ following LPS stimulation compared with adult cells [4]. Expression levels of regulatory cytokines such as IL-10 and transforming growth factor-β (TGF-β) have been documented to be limited in foals as well [4]. In contrast, basal and stimulus-induced levels of Th2-related cytokines such as IL-8, IL-12, and IL-23 have been shown to be comparable in foals and older individuals [3], [4]. Furthermore, in response to the specific pathogen *Rhodococcus equi*, no Th1-deficits have been found in neonatal foals [3], [6], [7] Thus, the basal and stimulus-induced cytokine expression in neonatal foals appears to be selectively impaired. Similarly, in human infants, who are biased towards Th2 responses as well, mature Th1 responses have been documented in response to Group B Streptococci [1].

Bacterial recognition by Toll-like receptors (TLRs) and the subsequent induction of signalling cascades have been shown to play a pivotal role in the functional defence against pathogens, as well as tolerance to commensal microorganisms [8], [9]. The induction of cytokine production by TLRs is crucial for both the innate immune response itself, and the initiation of adaptive immune responses [10]. Whereas TLR-4 responds to Gram-negative bacteria by sensing LPS, TLR-2 mainly recognises components of Gram-positive bacterial cell walls such as lipoproteins, lipoteichoic acid, and lipopeptides [8]. TLR-2 interacts with bacterial lipopeptides either independently by formation of TLR-2 homodimers, or in concert with TLR-1 or TLR-6 by formation of heterodimers [11]. TLR-5 and TLR-9 are activated by bacterial flagellin and specific bacterial DNA motifs, respectively, derived from both Gram-positive and Gram-negative bacteria [12], [13].

Dysfunction of TLRs has been associated with the occurrence of various inflammatory disorders in mammals, amongst others an increased susceptibility to bacterial infections and sepsis [14], [15]. Several *in vivo* studies in experimental animals have indicated that activation of certain TLRs, in particular TLR-2, can result in suppression of inflammatory pathology [16]–[18]. In the horse, limited research has been performed in this area, mainly focusing on TLR-9 ligands. *Ex vivo* activation of TLR-9 in peripheral blood mononuclear cells (PBMCs) isolated from neonatal foals has been shown to enhance the Th1 response [3]. However, in another study, antigen-presenting cells derived from foals did not respond to TLR-9 activation with increased expression of cytokines [19]. Likewise, *in vivo* administration of a TLR-9 ligand in foals did not result in a modulation of cytokine expression patterns [6]. Effects of TLR-2 activation on basal or stimulus-induced immune responses in the horse have not yet been described. Current research into immunomodulation focuses either on enhancement of TLR-dependent defensive immune responses, or suppression of excessive TLR activity, leading to inflammation and tissue damage. Both scenario's could apply to immunomodulation in horses, particularly in foals, and could contribute to lowering susceptibility to early, often life-threatening infections.

Hence, the aim of the current study was to explore the immunomodulatory properties of TLR-2 ligands in foals and adult horses. For this purpose, we used PBMCs from adult horses and newborn foals (<12 hours *postpartum*) to investigate the influence of TLR-2 activation on the LPS-induced inflammatory response. We investigated protein levels of TNF-α and IL-10, and mRNA expression levels of TNF-α and IL-6 (Th1-related cytokines), IL-10 (regulatory cytokine), and TLR-2, TLR-4, and TLR-9 (TLRs involved in bacterial recognition, for which the genetic sequence is known in the horse). Data were analysed with a Bayesian hierarchical regression model, accounting for variation between horses.

## Materials and Methods

### Animals and sample collection

Six healthy adult mares and foals (NRPS and New Forest breed) were sampled during this study. Foals were sampled within 12 hours postpartum. 60 ml of blood was collected by jugular venipuncture directly into sterile heparinised blood collection tubes (BD Vacutainer Systems, Plymouth, United Kingdom). Blood samples were kept cooled during transport to the laboratory, where PBMC isolation started within 2 hours after collection. All experimental procedures were approved by the committee of ethical considerations in animal experiments of Utrecht University (DEC Utrecht, Permit Number: 2011.II.06.101).

### PBMC isolation

Blood samples were diluted 1∶1 in fresh PBS (Lonza, Basel, Switzerland) containing 2 mM EDTA (Sigma- Aldrich, St. Louis) and subsequently layered over Ficoll-Paque™ plus (GE Healthcare, Waukesha, USA). After centrifugation (400× *g*, 30 minutes at room temperature) PBMCs were pipetted from the Ficoll layer and washed twice in PBS/EDTA. PBMCs were resuspended in RPMI 1640 Medium (Lonza, Basel, Switzerland) containing 2 mM glutamine (Lonza, Basel, Switzerland), 100 IU/ml penicillin (Lonza, Basel, Switzerland), 100 µg/ml streptomycin (Lonza, Basel, Switzerland) and 10% horse serum (prepared in our own laboratory by aseptically collecting blood, allowing clotting, and centrifuging). PBMCs were counted using trypan blue and resuspended to a density of 4*10^6^ cells/ml medium. Following storage overnight at 4°C to attenuate possible stimulatory effects of the applied Ficoll, PBMCs were seeded in 24 well plates at a density of 4*10^6^ cells/ml medium/well.

### Cell culture experiments

After seeding the PBMCs in 24 well plates, the plates were incubated for 2 hours at 37°C and 5% CO_2_. Thereafter, the plates were centrifuged for 10 minutes at 400× *g* before refreshing the medium without removing PBMCs. Before starting the experiments, PBMCs were pre-incubated for 2 hours with subculturing medium containing 0 or 1 µg/ml Pam3-Cys-Ser-Lys4 (PCSK; Invivogen, San Diego, USA), a synthetic bacterial lipopeptide that specifically activates TLR-2. After pre-incubation, the experiments were started by replacing the medium with medium containing 0 or 1 µg/ml LPS (*Escherichia coli* O111:B4; Sigma- Aldrich, St. Louis, USA) and 0 or 1 µg/ml PCSK. Plates were placed in the incubator and samples for qPCR and ELISA were taken after 4 hours. Each combination of incubations was investigated in triplicate for each horse. For the ELISAs, supernatants were stored at −80°C. For qPCR analyses, the PBMCs were lysed using RNA lysis buffer (Promega, Madison, USA) and stored at −80°C until RNA isolation was resumed.

### Cytokine measurements

To measure protein levels of TNF-α and IL-10, ELISA was performed on the supernatants using Duoset® ELISA Development System for equine TNF-α and equine IL-10 (R&D Systems, Minneapolis, USA). Standard operating procedures of the manufacturer were followed, applying all required buffers and solutions in the form provided by the manufacturer (R&D Systems, Minneapolis, USA). The detection limits of the ELISAs were 15.6 pg/ml (TNF-α) and 156.3 pg/ml (IL-10), respectively.

### RNA Isolation

RNA was isolated from PBMCs using SV Total RNA isolation system (Promega, Madison, USA) according to the manufacturer's instructions. Isolated fractions were dissolved in 50 µl RNAse free water and stored at −80°C. Quality and quantity of RNA was determined spectrophotometrically (Nanodrop).

### Real-time PCR analysis

cDNA was generated using iScript™ cDNA Synthesis Kit (Biorad, Hercules, USA) according to the manufacturer's protocol. For reverse transcriptase reaction, 1000 ng RNA was applied per sample. Expression of mRNA was assessed by real-time PCR using a Biorad iQ5 Multicolor Real-time PCR detection system and iQ™ SYBR® Green Supermix (Biorad, Hercules, USA). Specific primer pairs were designed and tested for efficiency and accuracy, after having checked their specificity using the NCBI-BLASTN search program. Primer pairs were synthesised commercially (Eurogentec, Maastricht, The Netherlands). For this study, mRNA expression of TLR-2, TLR-4, TLR-9, IL-6, IL-10, TNF-α, β-actin, and GAPDH was determined using the following primer pairs:

**TLR-2:** F 5′-TGCTGCCATTCTCATTCTTC-3′ R 5′-GGGCCACTCCAGGTAGGT-3′;

**TLR-4:** F 5′-CCCTTTCAACTCTGCCTTCACT-3′ R 5′-GGGACACCACGACAATAACTTTC-3′;

**TLR-9:** F 5′-GACTGGCTACCTGGCAAGAC-3′ R 5′-GAAGCTGGCACGCAAGAG-3′;

**IL-6:** F 5′-TGGCTGAAGAACACAACAACT-3′ R 5′-GAATGCCCATGAACTACAACA-3′;

**IL-10:** F 5′-GAGAACCACGGCCCAGACATCAAG-3′ R 5′-GACAGCGCCGCAGCCTCACT-3′;

**TNF-α:** F 5′-TCCAGACGGTGCTTGTGC-3′ R 5′-GGCCAGAGGGTTGATTGACT-3′;

**β-actin:** F 5′-CAAGGCCAACCGCGAGAAGATGAC-3′ R 5′-GCCAGAGGCGTACAGGGACAGCA-3′;

**GAPDH:** F 5′-TGGCATGGCCTTCCGTGTCC-3′ R 5′-GCCCTCCGATGCCTGCTTCAC-3′.

### Data analysis and statistical methods

Data from the second time point (4 hours incubation) were analysed in a Bayesian hierarchical linear regression model. Log-transformed ELISA results (log-concentrations) and qPCR results (cycle threshold value or Ct value) were assumed to be normally distributed. For all incubations (controls, LPS, PCSK, or LPS+PCSK) population means of the log-concentrations and Ct values were modelled in mares and foals, allowing responses to differ between mares and foals. Inter-individual variation was modelled by means of an error term at the level of horses (i.e. assuming that PBMCs from some horses are more ‘reactive’ in general than PBMCs from others) and incubation types within horses (i.e. assuming that horse PBMCs may react differently to specific incubations). Because TNF-α ELISA signals of all control samples were below the detection limit, we assumed signal values equal to the detection limit, leading to a conservative estimate of differences between controls and other samples. Because IL-10 ELISA signals were mostly above the detection limit, those few values that were below the detection limit (n = 2) were left-censored; i.e. assuming that the ‘true’, unobserved IL-10 concentration is such that the ‘observed’ value is likely to be under the detection limit.

Analyses were performed in JAGS, a program for analysis of Bayesian models using Markov Chain Monte Carlo (MCMC) simulation (version 3.2.0) [20]. Simulations in JAGS were set up and analysed in R (version 2.14.2)[21], using packages *rjags* (version 3–5) [22] and *R2jags* (version 0.03–06) [23]. Posterior distributions were estimated based on uninformative prior distributions (normal distributions with mean 0 and standard deviation 100 for parameter means; inverse gamma distributions with mean 1 and variance 10,000 for parameter variances). Bayesian credible intervals (BCI) for parameter estimates were based on the 2.5% and 97.5% percentiles of posterior distributions. Posterior distributions were simulated by means of four Markov chains, each consisting of 30,000 Monte Carlo samples. The first 10,000 samples were discarded for burn-in, allowing the model to converge. Model convergence was assessed by checking whether chains converged to the same posterior distribution, based on Gelman and Rubin's convergence diagnostic, the potential scale reduction factor [24].

Differences were stated significant, based on the calculated 95% BCI. In the results section, significant differences are quantified by factors or percentages, though not all significant differences are quantified in the results section. An overview of all data and the corresponding 95% BCI's is given in an additional file, annexed to the manuscript (“Data S1”).

### qPCR data validation

Variation of mRNA expression was excluded as a confounding factor, as no significant differences in either β-actin or GAPDH expression were detected amongst control samples and the incubations with LPS, PCSK, and LPS+PCSK.

In addition, we analysed the data with a correction for these housekeeping genes, according to the method described by Livak and Schmittgen [25]. In our case, normalisation of data (based on housekeeping genes) did not change point estimates for differences between incubation types. Furthermore, normalisation did not reduce the variation in the data (as should be expected in case of confounding due to variation in total mRNA levels). This indicated that the error due to variation in mRNA expression, if any, was negligible compared to basic measurement error in the data. Based on this knowledge and the fact that housekeeping genes for equine PBMCs have not yet been validated, we decided to present the data in this manuscript as they are (i.e. not normalised).

## Results

### Cytokine production

**Figure 1:**
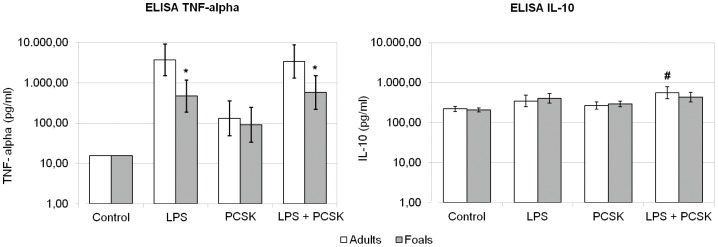
ELISA results for TNF-α and IL-10 in adult and neonatal PBMCs. Mean concentrations of cytokines (pg/ml) and 95% Bayesian credible intervals are included for PBMCs incubated with blank medium (Control), with 1 µg/ml LPS, with 1 µg/ml Pam3-Cys-Ser-Lys4 (PCSK), or with a combination of both LPS and PCSK. Compared to control samples, there was a significant increase of both TNF-α and IL-10 in response to all tested compounds in both groups (not indicated in the figure). Significant differences between responses in adults and foals are indicated with an asterisk (*). A significant difference between the LPS response and the response to LPS+PCSK (within the same age group) is indicated with a hash (#).

ELISA results for equine TNF-α and IL-10 are illustrated in figure 1. In comparison with controls, all tested conditions (LPS, PCSK, and LPS+PCSK) led to a significant increase of TNF-α and IL-10 production by PBMCs of both adult and newborn horses (not indicated in the figure). In both age groups, TNF-α production by PBMCs after LPS stimulation was not significantly influenced by pre-incubation and co-stimulation of the cells with PCSK. In contrast, IL-10 production by adult PBMCs challenged with LPS increased significantly (1.6-fold) when the cells were co-incubated with PCSK. In foals, this effect was not significant. Compared with adult PBMCs, foal PBMCs produced significantly lower amounts of TNF-α after LPS stimulation (87% lower) and after incubation with both LPS and PCSK (83% lower).

### Cytokine expression

**Figure 2:**
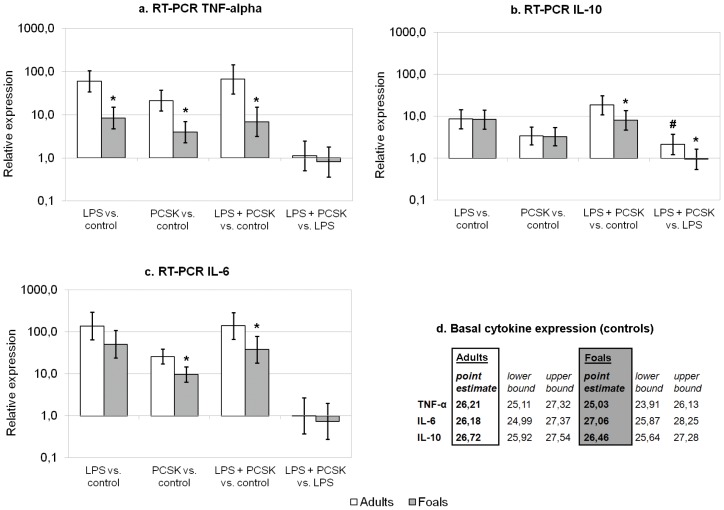
qPCR results for TNF-α, IL-6, and IL-10 in adult and neonatal PBMCs. In Figure 2a, 2b, and 2c, mean values and 95% Bayesian credible intervals are given for relative expression levels (compared to controls and LPS stimulated cells) in PBMCs incubated with blank medium (Control), 1 µg/ml LPS, with 1 µg/ml Pam3-Cys-Ser-Lys4 (PCSK), or with a combination of both LPS and PCSK. Compared to control samples, there was a significant increase of both TNF-α, IL-6, and IL-10 in response to all tested conditions in both age groups (not indicated in the figure). In Figure 2d, estimated means for Ct values and 95% Bayesian credible intervals are stated for basal cytokine expression levels in both groups. Significant differences between responses in adults and foals are indicated with an asterisk (*). A significant difference between the LPS response and the response to LPS+PCSK (within the same age group) is indicated with a hash (#).

In figure 2, the qPCR results are summarized for equine TNF-α, IL-6, and IL-10. Basal expression levels of these cytokines did not differ significantly between the two age groups (figure 2d). In both age groups, expression of all measured cytokines was relatively higher for incubations with LPS, PCSK, or both, compared with control samples, similar to cytokine protein levels (not indicated in the figure). Furthermore, in both age groups, TNF-α and IL-6 expression in response to LPS was not significantly influenced by co-stimulation with PCSK. However, in adult PBMCs, incubation with LPS and PCSK resulted in a significant increase in IL-10 expression (2.2-fold), whilst in neonatal PBMCs this effect was not significant. Relative expression of TNF-α in foal PBMCs was significantly lower compared to adult PBMCs in all incubations (86% lower in foal PBMCs incubated with LPS, 81% lower for PCSK, and 90% lower for LPS+PCSK). In addition, relative expression of IL-6 was significantly lower in foal PBMCs incubated with PCSK (63% lower) and LPS+PCSK (73% lower). For IL-10, relative expression was only significantly different between adult and foal PBMCs incubated with LPS and PCSK (57% lower in foals).

### TLR expression

**Figure 3:**
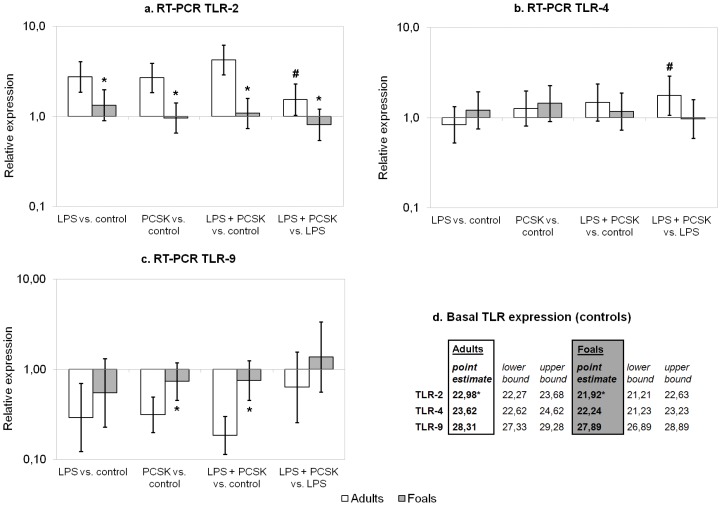
qPCR results for TLR-2, TLR-4, and TLR-9 in adult and neonatal PBMCs. In Figure 3a, 3b, and 3c, mean values and 95% Bayesian credible intervals are given for relative expression levels (compared to controls and LPS stimulated cells) in PBMCs incubated with blank medium (Control), 1 µg/ml LPS, with 1 µg/ml Pam3-Cys-Ser-Lys4 (PCSK), or with a combination of both LPS and PCSK. In adult PBMCs, significant up-regulation of TLR-2 expression and significant down-regulation of TLR-9 was found in response to LPS, PCSK, and LPS+PCSK (not indicated in the figure). In Figure 3d, estimated means for Ct values and 95% Bayesian credible intervals are stated for basal TLR expression levels in both groups. Significant differences between responses in adults and foals are indicated with an asterisk (*). A significant difference between the LPS response and the response to LPS+PCSK (within the same age group) is indicated with a hash (#).

Figure 3 summarises RT-PCR results for TLR-2, TLR-4, and TLR-9. In control samples, TLR-2 expression was significantly higher in foals (2.1-fold) in comparison with adults (average Ct values were 22.98 for adult PBMCs, and 21.92 for foal PBMCs). Basal expression of TLR-4 and TLR-9 did not differ significantly between the two age groups (Figure 3d). Though, the difference in basal expression of TLR-4 between the two age groups was very close to being significant. Mean Ct values for TLR-4, calculated from this model for adult and foal PBMCs, were 23.62 and 22.24 respectively (average ΔCt = 1.39, 95% BCI: −0.02–2.80), indicating a factor 2.6 difference in expression level.

In foal PBMCs, no significant change in the expression of TLR-2, TLR-4, or TLR-9 was found in response to LPS, PCSK, or LPS+PCSK in comparison with control samples. In contrast, in adult PBMCs there was a significant up-regulation of TLR-2 expression and a significant down-regulation of TLR-9 in response to all tested TLR ligands, whereas TLR-4 expression did not change significantly in comparison to controls. Furthermore, TLR-2 expression was relatively lower in foal PBMCs compared to adult PBMCs under all tested conditions (51% lower in foal PBMCs incubated with LPS, 64% for PCSK, and 75% for LPS+PCSK). TLR-9 expression was relatively higher in foal PBMCs incubated with PCSK (2.3-fold) and LPS+PCSK (4.1-fold) compared to adult PBMCs. In adult PBMCs, a significant increase of TLR-2 and TLR-4 expression was found after incubation with LPS+PCSK compared to LPS alone (1.6-fold increase for TLR-2, 1.8-fold for TLR-4). In contrast, in LPS challenged foal PBMCs, pre-incubation and co-stimulation with PCSK did not lead to changes in TLR-2 or TLR-4 expression levels. TLR-9 expression in response to LPS was not significantly influenced by co-stimulation with PCSK in either group.

## Discussion

### Cytokine production and expression

Our data show that the TNF-α response in foal PBMCs following LPS stimulation do not reach the level of adult PBMCs. This finding is in line with previously published results, demonstrating that in newborn foals, the cytokine response to external challenges like LPS, including Th-1 related and regulatory cytokines, is lower than that of adult individuals [1]–[3]. However, Mérant et al [4] documented comparable TNF-α expression levels after LPS challenge in foal and adult MoDCs. Most likely, this apparent discrepancy is caused by the difference in age of the tested foals. Mérant et al [4] sampled 2–3 weeks old animals, whereas the study population in the experiment described here comprised true neonates, sampled within 12 hours *postpartum*. The limited expression and production of IL-10 we documented in LPS challenged foal PBMCs compared to adult PBMCs was consistent with the limited IL-10 mRNA expression levels in LPS challenged MoDCs derived from foals [4]. Apparently, in the foal, the TNF-α response matures earlier in life compared with the IL-10 response.

In our experimental model, TNF-α production after LPS challenge was not significantly influenced by pre-incubation and co-stimulation with PCSK in either age group. On the other hand, TLR-2 stimulation in addition to TLR-4 activation resulted in a significant increase of IL-10 production in adult PBMCs (1.6-fold). In foals, this effect was not observed. This increase of IL-10 production (and not TNF-α) as a result of concomitant TLR-2 stimulation fits the hypothesis that TLR-2 ligands exhibit anti-inflammatory properties, as the secretion of IL-10 activates regulatory T cells, which subsequently will produce IL-10 and TGF-β. As a result of these cytokines, Th1, Th2, and Th17 responses are suppressed [26], [27].

Regarding the differences in cytokine production between PBMCs incubated with TLR-2 or TLR-4 ligands, our results are in agreement with the existing literature on mammalian species [28], [29]. We found that TLR-2 activation resulted in a relatively mild inflammatory response, whereas TLR-4 activation triggered a marked inflammatory response, characterised by a high peak of TNF-α production after 4 hours of incubation with LPS. This pattern was evident in both adult and foal PBMCs.

### TLR expression

TLR expression levels in foals were stable under all different experimental conditions. In contrast, in adult PBMCs, significant up-regulation of TLR-2 and down-regulation of TLR-9 were evident after both separate and concomitant TLR-2 and TLR-4 activation. Expression of TLR-4 did not change significantly. Besides clear differences in the regulation of TLR expression levels between PBMCs derived from newborn and adult horses, these qPCR results also demonstrate TLR crosstalk in this experimental model, as the expression of TLR-2 and TLR-9 was significantly influenced by both TLR-2 and TLR-4 activation. Remarkable was that though TLR expression rates in foal PBMCs were not influenced by the used TLR ligands (in contrast to adult PBMCs), basal TLR expression levels in foal PBMCs tended to be higher than in adult PBMCs.

Our observation of coinciding lower cytokine responses and higher TLR expression levels in PBMCs derived from neonatal foals suggests that TLR signalling is impaired in newborn foals compared with adult horses. Moreover, our data illustrate that in foals, TLR expression levels are not influenced by a challenge with bacterial patterns, representing either Gram-negative or Gram-positive bacteria, in contrast to adult horses. Such a limited TLR function is essential in neonates, as the transition from the sterile womb into an environment full of micro-organisms would lead to exaggerated inflammatory responses and subsequent pathology if all TLR signalling pathways were fully operational. Tolerance to commensal flora is a prerequisite for bacterial colonisation of epithelial surfaces including the gut. The relatively high TLR expression levels, which were found in foals, might facilitate more profound defensive responses towards specific pathogens, if necessary. For instance, *Rhodococcus equi* is a specific pathogen that elicits mature defensive responses in young foals [6], [7]. The challenge for future research in this area is to find methods or ligands that enhance TLR functionality and bacterial defence mechanisms in young foals without interfering with the physiological process of bacterial tolerance and colonisation.

### Ligation of TLR-2 by different compounds

PCSK is a confirmed TLR-2 agonist, which is often applied in the published literature throughout the past decades. Nonetheless, other compounds have been described as TLR-2 ligands as well. PCSK binds to TLR-2/TLR-1 heterodimers and does not interact with TLR-6, unlike other TLR-2 agonists [30]. Long et al [31] compared effects of four different TLR-2 agonists, including PCSK. Though all tested TLR-2 ligands were shown to activate NFκB and MAPK signalling pathways in murine macrophages, the degree of activation differed among the tested compounds. Moreover, significant differences were documented in cytokine responses as well as the *in vivo* recruitment of leukocytes in a murine model of acute inflammation. Thus, although we can conclude that TLR-2 was ligated in our model, we cannot exclude that other TLR-2 agonists would possibly modulate the inflammatory responses in this equine model differently.

## Conclusion

The obtained data demonstrate a distinct regulation of cytokine and TLR expression levels in foal and adult PBMCs in response to TLR-2 and TLR-4 activation. While TLR expression levels were high in foals, the lower functional response following TLR activation in neonatal foals can be considered as a strategy to limit the immune responses to the bacterial colonisation of epithelial surfaces during the first period of life. In turn, the relatively high TLR expression levels in foals might be responsible for the previously described marked immune responses to specific pathogens, such as *Rhodococcus equi*. PCSK, which has been shown to alter inflammatory responses beneficially in previously studied experimental models by activating TLR-2, did not exhibit comparable properties in our model. Hence, PCSK does not seem to be a promising candidate for immunomodulation in foals. This indicates that translation of findings from one animal species to another is limitedly possible and requires confirmation. It remains a challenge for future research to identify substances or methods, which enhance TLR functionality and bacterial defence in young foals without causing an exaggerated inflammatory response or interfering with bacterial colonisation.

## Supporting Information

Data S1 **Parameter estimates and 95% Bayesian credible intervals for all investigated parameters.**
(XLSX)
